# Who’s opting-in? A demographic analysis of the U.K. NHS Organ Donor Register

**DOI:** 10.1371/journal.pone.0209161

**Published:** 2019-01-02

**Authors:** Catrin Pedder Jones, Chris Papadopoulos, Gurch Randhawa

**Affiliations:** Institute for Health Research, University of Bedfordshire, Putteridge Bury, Luton, United Kingdom; University of Cambridge, UNITED KINGDOM

## Abstract

The NHS Organ Donor Register (NHS ODR) is a centralised database for U.K. residents wishing to be organ donors. Opt-in membership to the NHS ODR demonstrates an expression of a wish to donate, which can be key in decisions made by family members at time of death. By examining the demographic breakdown of the 24.9 million registrants, campaigns can be better targeted to increase membership among those groups underrepresented on the NHS ODR. Data from the NHS ODR (as of March 2017) was analysed using Chi^2^ Goodness of Fit analyses and Chi^2^ Test of Independence for the categorical variables of gender, nation of residency at time of registration, ethnicity, organ preference, registration age and age at registration. Goodness of fit analyses showed significant differences between demographic representation on the NHS ODR compared to the U.K. population. Cramer’s V showed significant associations were only of note (above 0.1) for age, ethnicity in the U.K. as a whole and ethnicity in England. Older (70+) and younger people (0–14) were underrepresented and those of White Ethnicity overrepresented on the NHS ODR. Although association strength was weak, more women and less residents of England were present compared to the U.K. population. Tests of independence showed significant differences between age at registration and current age on the register and cornea donation preferences. These results indicate areas for targeting by campaigns to increase NHS ODR membership. By understanding the strength of these associations, resources can be utilised in areas where underrepresentation is larger and will have the most impact to demographics of the NHS ODR. Additionally, by identifying which groups are over and underrepresented, future research can explore the reasons for this in these demographic groups.

## Introduction

The United Kingdom needs more organ donors. Between April 2017 and March 2018 1,574 people became deceased organ donors, resulting in 4,039 transplants [1]. During this same period however 6,044 people were on the active transplant waiting list and 426 people died whilst waiting for an organ [1]. Deceased organ donation involves the removal of a donor’s organs; these are then transplanted into a recipient in need. In the UK, consent for deceased organ donation occurs through NHS staff consulting with the donor’s next of kin, who ultimately makes the decision [1]. Within the UK (England, Scotland, Wales and Northern Ireland), a system of organ donation exists where people can express their wishes to opt-in to organ donation in England, Scotland and Northern Ireland; and opt-in or opt-out in Wales—through registering with a centralised database [2,3]. This is the NHS Organ Donor Register (NHS ODR) run by NHS Blood and Transplant.

Set up in 1994, the NHS ODR allows people to register their intention to become an organ donor and helps guide family members in their decision making [4]. As part of this process, a person can choose to donate all their organs or be selective over which they choose to donate [1]. At the end of March 2018, there were 24.9 million people on the NHS ODR, equating to 38% of the U.K. population [1]. Prior expression of a persons’ wish to donate is found to be a strong predictor of a positive donation decisions by families [5–7]. Research has found that the decision making process for families is less complex and involves fewer cognitive factors when a person’s wishes are known [8]. The significance of this is clear in the U.K as 48% of donors in the U.K. were registered on the NHS ODR (1,574) [1].

A strategy, titled, ‘Taking transplantation to 2020’, has been developed that aims to match UK organ donation rates with the highest performing countries [9]. The objectives include increasing the number of people on the NHS ODR to at least 50% by 2020 and to encourage conversation using the NHS ODR as a tool [9]. The strategy also includes a long-term recommendation to ‘Develop an audience segmentation model and targeted direct marketing campaign to under-represented groups’ [9]. Additional elements of this strategy, however, emphasise the potential of low cost strategies to help achieve this objective [9]. Identifying which groups are underrepresented and the degree to which this underrepresentation exists should help towards achieving these goals.

NHS Blood and Transplant produce yearly activity reports for all organ donation and transplants carried out [1]. The NHS ODR report provides an overview of the breakdown of membership; by sign-up method, age group, gender, ethnicity and socioeconomic status. These activity reports help NHS Blood and Transplant (NHSBT) to target specific groups who are underrepresented on the NHS ODR and as donors. According to these reports, one of the least represented groups is those of Black, Asian and Minority Ethnicities (BAME), with BAME registrants making up just 7.4% of the NHS ODR between April 2017 and March 2018 [10]. These statistics are particularly concerning given that BAME populations made up 30% of the active transplant waiting list but only 23% of donor recipients [10]. They also typically wait approximately 6 months longer for a transplant than Non-BAME patients [10].

NHSBT activity reports are a valuable resource for examining demographic differences in donation and sign-up which can be useful in targeting campaigns to specific groups and sub-groups (for example, BAME groups). Despite this, no previous academic study has statistically investigated in detail the demographic profile of the NHS ODR. Analyses which compare demographic characteristics of the ODR to those in the population will allow specific targeting to groups that are underrepresented. Thus, the first aim of this study was to answer the question: How does demographic membership of the NHS Organ Donor Register differ from the demographic patterns in the U.K.?

Although this first aim is important, understanding the demographic patterns in comparison to the U.K. general population alone may not be enough to suitably target interventions and provide an in depth understanding of demographic patterns in the NHS ODR. Therefore, analysis of the relationships between these variables will be conducted to investigate the second question; how do the demographic variables on the NHS ODR relate to each other?

## Method

### Ethical approval

An application was made to NHSBT for the supply of data using the UK Transplant Registry–Access to Data form in November 2016. The anonymised and de-personalised data was provided to the research team in person via encrypted memory stick. Once NHSBT process and approval was completed an application was made to the University of Bedfordshire Research Ethics Committee, with approval to commence analyses provided in April 2017 (Approval Number IHREC729).

### Dataset and sample

The two datasets were provided by NHSBT and contained anonymised versions of the NHS ODR. Dataset 1 is a freeze of the NHS ODR as of April 2015, it includes the variables Date of registration, age at registration, current age (as of April 2015), gender, ethnicity, source of registration and nation. Dataset 2 is the current NHS ODR as of March 2017 which includes the variables in dataset 1, except ethnicity and includes organ donation preference (whether the registrant has elected to donate all their organs or selected organs). Prior to analyses, age at registration and current age were converted into categorical age groups (0–4, 5–9, 10–14, 15–19, 20–24, 25–29, 30–34, 35–39, 40–44, 45–49, 50–54, 55–59, 60–64, 65–69, 70–74, 75–79, 80–84, 85–89, 90+). These groups were selected to reflect the existing age groups used by the Office of National Statistics (ONS).

### Analyses

Two stages of analyses were conducted; a comparison to the U.K. population and tests of association between demographics. For stage 1 Chi^2^ Goodness of Fit tests were used to compare demographic membership of the Organ Donor Register with demographic percentages in the general population. The variables gender, ethnicity, age group at registration, current age group and nation were analysed in this way assuming unequal proportions. For stage 2 the second set of tests were Chi^2^ test of independence. These examined whether a significant relationship exists between two different demographic variables and where exactly within the variables these lie (e.g. men from England are significantly more likely than women from Scotland to sign-up to the NHS ODR). These were also conducted for nation, gender, ethnicity, registration age and current age. Additionally, preference to donate individual organs was included in this analyses to examine whether this differs according to demographic categories.

A key consideration of these analyses however is the very large sample size. More than 23 million people are members of the NHS ODR in this dataset, therefore due to test sensitivity it is likely most Chi^2^ tests will return a significant result. To overcome this Cramer’s V was used to measure the strength of association in Chi^2^ tests. Scores of 0.1 are considered a small effect, 0.3 a medium effect and 0.5 a large effect. If Cramer’s V is below 0.1 the association is considered to be too small to be worthy of attention statistically [11]. All analyses were conducted using SPSS V22.

Missing data analyses were conducted for all variables, and where ‘unknown’ or ‘not reported’ were provided by NHSBT, these were included in the missing data frequencies. In the March 2017 dataset all variables had less than 1% missing data; current age 0.03%, registration age 0.88%, gender 0.29%, nation 0.28%. In the pre April 2015 dataset however ethnicity had 76.96% missing data. NHSBT has previously reported that analysis of ethnicity in their activity reports is problematic due to this and suggests the high level of missing data is due to ethnicity not being recorded via some methods of sign-up and where it is recorded it is not a mandatory field for completion.

## Results

The results are split by the dataset in which they were analysed. Dataset 1 contains the most recent version of the NHS ODR (March 2017) the variables gender, nation and age were analysed in this dataset. Ethnicity data was analysed using the dataset frozen as of April 2015, dataset 2. It is important however to consider that direct comparisons between dataset 1 and dataset 2, may not be appropriate as demographic proportions of the NHS ODR could have changed in the time period between April 2015 and March 2017. To examine if significant changes in demographics occurred between the frozen 2015 dataset 2 and the current March 2017 dataset 1, a Chi^2^ goodness of fit test was used to compare the % of each gender, age group at registration, current age group and nation between the two datasets. All results indicate a significant Chi^2^, however all have a very small Cramer’s V under .1, indicating the difference between the datasets is of weak strength of association and not statistically worthy of attention (Gender X^2^(1) 35.183 p < .001 Cramer’s V = .001; age group at registration X^2^(8)9166.743 p < .001, Cramer’s V = .01; current age group X^2^(8) 64544.724 p < .001, Cramer’s V = .02; nation X^2^(4) 3318.450 p < .001, Cramer’s V = .01). These results indicate it is appropriate to use both dataset 1 and dataset 2 for our analyses and the results are unlikely to be influenced by significant demographic differences between the time points.

Table 1 displays the demographic distribution of the NHS ODR compared to the general U.K. population [12,13]. This table shows that less males, people of Asian, Black, Chinese, Mixed and Other ethnicity and people currently aged 0–24 and 75–90+, are registered on the NHS ODR than present in the general U.K. population.

**Table 1:** Demographic distribution of NHS ODR compared with the U.K. general population.

		NHS ODR N (%)	U.K. Population N (%) *
**Gender^**	**Male**	10830106 (46.5%)	32377674 (49.3%)
	**Female**	12473804 (53.5%)	33270380 (50.7%)
**Nation^**	**England**	19060896 (81.6%)	55268100 (84.2%)
	**Wales**	1157582 (5.0%)	3113200 (4.7%)
	**Scotland**	2365021 (10.1%)	5404700 (8.2%)
	**Northern Ireland**	723338 (3.1%)	1862100 (2.8%)
**Registration Age^**	**0–4**	348632 (1.5%)	4,014,314 (6.1%)
	**5–9**	163894 (0.7%)	4,037,456 (6.2%)
	**10–14**	244110 (1.0%)	3,625,062 (5.5%)
	**15–19**	4416524 (18.9%)	3,778,927 (5.8%)
	**20–24**	2460251 (10.5%)	4,253,751 (6.5%)
	**25–29**	2855796 (12.2%)	4,510,648 (6.9%)
	**30–34**	2373948 (10.2%)	4,408,163 (6.7%)
	**35–39**	2007287 (8.6%)	4,179,537 (6.4%)
	**40–44**	1755326 (7.5%)	4,174,065 (6.4%)
	**45–49**	1584631 (6.8%)	4,619,147 (7.0%)
	**50–54**	1342209 (5.7%)	4,631,981 (7.1%)
	**55–59**	1073767 (4.6%)	4,066,685 (6.2%)
	**60–64**	832983 (3.6%)	3,534,233 (5.4%)
	**65–69**	978176 (4.3%)	3,636,517 (5.5%)
	**70–74**	531163 (2.3%)	2,852,065 (4.3%)
	**75–79**	237967 (1.0%)	2,154,524 (3.3%)
	**80–84**	78709 (0.3%)	1,606,746 (2.4%)
	**85–89**	24250 (0.1%)	992,988 (1.5%)
	**90+**	61595 (0.3%)	571,245 (0.9%)
**Current Age^**	**0–4**	82088 (0.4%)	4,014,314 (6.1%)
	**5–9**	110062 (0.5%)	4,037,456 (6.2%)
	**10–14**	108830 (0.5%)	3,625,062 (5.5%)
	**15–19**	593158 (2.5%)	3,778,927 (5.8%)
	**20–24**	1470847 (6.3%)	4,253,751 (6.5%)
	**25–29**	2031314 (8.7%)	4,510,648 (6.9%)
	**30–34**	2256780 (9.7%)	4,408,163 (6.7%)
	**35–39**	2472607 (10.6%)	4,179,537 (6.4%)
	**40–44**	2317232 (9.9%)	4,174,065 (6.4%)
	**45–49**	2419899 (10.4%)	4,619,147 (7.0%)
	**50–54**	2157589 (9.2%)	4,631,981 (7.1%)
	**55–59**	1732592 (7.4%)	4,066,685 (6.2%)
	**60–64**	1393202 (6.0%)	3,534,233 (5.4%)
	**65–69**	1255913 (5.4%)	3,636,517 (5.5%)
	**70–74**	1164751 (5.0%)	2,852,065 (4.3%)
	**75–79**	758709 (3.2%)	2,154,524 (3.3%)
	**80–84**	523867 (2.2%)	1,606,746 (2.4%)
	**85–89**	311394 (1.3%)	992,988 (1.5%)
	**90+**	210384 (0.9%)	571,245 (0.9%)
**Ethnicity^^**	**White**	639524 (93.8%)	55,010,359 (87.1%)
	**Asian**	19220 (2.8%)	3,940,189 (6.2%)
	**Black**	5774 (0.8%)	1,904,684 (3.0%)
	**Chinese**	1934 (0.3%)	433,150 (0.7%)
	**Mixed**	12068 (1.8%)	1,250,229 (2.0%)
	**Other**	3409 (0.5%)	643,567 (1.0%)
**Positive Consent to Donate Organ^**	**Kidney**	22150786 (99.2%)	N/A
	**Pancreas**	21754521 (97.4%)	N/A
	**Heart**	21739258 (97.3%)	N/A
	**Lungs**	21784412 (97.5%)	N/A
	**Liver**	22003939 (98.5%)	N/A
	**Cornea**	19957594 (89.3%)	N/A

^ Analysed using dataset 1 –March 2017.

^^Analysed using dataset 2 –April 2015.

*U.K. population data from Office of National Statistics: gender, age, nation extracted from 2016 population estimate data [12]. Ethnicity data extracted from 2011 census data [13].

### Gender

The data was examined to determine how NHS ODR membership compares to the gender distribution in the entire U.K. population. According to the ONS the percentage of males and females in the U.K. in 2016 was 49.3% and 50.7% respectively [12]. A Chi^2^ Goodness of fit test was found to be significant, comparing the NHS ODR dataset to these percentages X^2^(1) = 74493.808 p < .001, with more females on the ODR than males compared to the U.K. population. The strength of this result was weak however (Cohen, 1988) with a Cramer’s V = .06.

### Nation

According to the ONS the population percentages of the U.K. are as follows; England 84.2%, Wales 4.7%, Scotland 8.2% and Northern Ireland 2.8% [12]. These were used in the Chi^2^ Goodness of fit test which was significant, X^2^(3) 134998.972 p < .001 with more Scottish registrations (residual 451,947.3), Northern Irish (residual 70,093.3) and Welsh (residual 61,064.1) than expected and less English registrations (residual -583,104.8). The strength of this result was also weak (Cohen, 1988) with a Cramer’s V = .04.

### Age groups

Two Chi Square Goodness of fit analyses were conducted for age groups, both were significant; registration age groups X^2^ (18) 13695513.8 p < .001, and current age groups X^2^ (18) 6146979.7, p < .001. The strength of this result achieved the threshold of note (.1) for Cramer’s V, V = .18 and V = .12 respectively. When examining where these significant differences lie for registration age, Table 2 shows that fewer people aged 0–14 and aged 45–95 register on the NHS ODR than in the general population (Table 2). However more 25–74 year olds are present on the NHS ODR than expected and more people register at the ages of 15–44 than expected.

**Table 2:** Observed and expected frequencies for age group at registration and current age group compared to the U.K. general population (analysed using dataset 1 –March 2017).

Variable	Age Group	Observed N	Expected N
**Registration Age**	**0–4**	348632	1424220.1
	**5–9**	163894	1447567.9
	**10–14**	244110	1284132.9
	**15–19**	4416524	1354176.5
	**20–24**	2460251	1517611.6
	**25–29**	2855796	1611003.0
	**30–34**	2373948	1564307.3
	**35–39**	2007287	1494263.7
	**40–44**	1755326	1494263.7
	**45–49**	1584631	1634350.9
	**50–54**	1342209	1657698.8
	**55–59**	1073767	1447567.9
	**60–64**	832983	1260785.0
	**65–69**	978176	1284132.9
	**70–74**	531163	1003958.4
	**75–79**	237967	770479.7
	**80–84**	78709	560348.9
	**85–89**	24250	350218.1
	**90+**	61595	210130.8
**Current Age**	**0–4**	82088	1424220.1
	**5–9**	110062	1447567.9
	**10–14**	108830	1284132.9
	**15–19**	593158	1354176.5
	**20–24**	1470847	1517611.6
	**25–29**	2031314	1611003.0
	**30–34**	2256780	1564307.3
	**35–39**	2472607	1494263.7
	**40–44**	2317232	1494263.7
	**45–49**	2419899	1634350.9
	**50–54**	2157589	1657698.8
	**55–59**	1732592	1447567.9
	**60–64**	1393202	1260785.0
	**65–69**	1255913	1284132.9
	**70–74**	1164751	1003958.4
	**75–79**	758709	770479.7
	**80–84**	523867	560348.9
	**85–89**	311394	350218.1
	**90+**	210384	210130.8

### Ethnicity

ONS 2011 census data was used to compare the proportion of each ethnic group on the NHS ODR than those present in the general population (87.1% White, 6.2% Asian, 3% Black, 0.7% Chinese, 2% Mixed and 1% Other) [13]. The ethnic groups used were those provided by NHS Blood and Transplant in the pre-April 2015 dataset. A significant Chi^2^ Goodness of Fit was found X^2^(5) 380210.033 p < .001 for these ethic groups across the entire U.K. population. The strength of this result is also small and reaches the threshold for a notable Cramer’s V, .13. More people identifying as White are present on the NHS ODR than all other ethnic groups (Table 3).

**Table 3:** Observed and expected frequencies for ethnicity across entire UK (analysed using dataset 2 –April 2015).

		Observed	Expected
**Ethnicity**	**White**	639524	593960.2
	**Asian**	19220	42279.6
	**Black**	5774	20457.9
	**Chinese/Oriental**	1934	4773.5
	**Mixed**	12068	13638.6
	**Other**	3409	6819.3

When analysed by nation, significant Chi^2^ Goodness of Fit tests were found for Wales X^2^(5)4482.410 p < .001, Scotland X^2^(5) 10864.043 p < .001, England X^2^(5)367738.348 p < .001, and Northern Ireland X^2^(5) 954.532 p < .001. Only the result for England reached the Cramer’s V threshold of note (.1; Cohen, 1988), Wales V = .07, Scotland V = .07, England V = .13 and Northern Ireland V = .04. As found for the U.K., more people identifying as White were present on the NHS ODR in all nations than expected. However, more people identifying as Mixed ethnicity were present on the NHS ODR in Scotland and Northern Ireland than expected.

### Tests of association

#### Gender x age

A significant Chi^2^ test of independence was found for age groups at registration and gender X^2^ (18) 89218.893, p < .001. The association was very small [11], Cramer’s V = .06.

Significant results were also found for current age groups and gender X^2^ (18) 48558.306, p < .001, the association was also small [11] Cramer’s V = .05. Table 4 represents the adjusted residuals for these calculations.

**Table 4:** Chi^2^ test of independence adjusted residuals for gender and age groups (analysed using dataset 1 –March 2017).

		Female	Male
**Registration Age**	**0–4**	-55.6	55.6
	**5–9**	-35.5	35.5
	**10–14**	67.3	-67.3
	**15–19**	112.0	-112.0
	**20–24**	147.0	-147.0
	**25–29**	37.9	-37.9
	**30–34**	-7.2	7.2
	**35–39**	-32.6	32.6
	**40–44**	-32.7	32.7
	**45–49**	-16.6	16.6
	**50–54**	-13.7	13.7
	**55–59**	-31.7	31.7
	**60–64**	-46.4	46.4
	**65–69**	-161.1	161.1
	**70–74**	-104.3	104.3
	**75–79**	-76.4	76.4
	**80–84**	-36.3	36.3
	**85–89**	-12.4	12.4
	**90+**	38.0	-38.0
**Current Age**	**0–4**	-26.5	26.5
	**5–9**	-31.5	31.5
	**10–14**	-24.3	24.3
	**15–19**	55.1	-55.1
	**20–24**	76.3	-76.3
	**25–29**	68.9	-68.9
	**30–34**	67.7	-67.7
	**35–39**	43.7	-43.7
	**40–44**	33.2	-33.2
	**45–49**	1.0	-1.0
	**50–54**	-27.8	27.8
	**55–59**	-36.1	36.1
	**60–64**	-40.1	40.1
	**65–69**	-38.8	38.8
	**70–74**	-68.6	68.6
	**75–79**	-82.0	82.0
	**80–84**	-89.5	89.5
	**85–89**	-55.5	55.5
	**90+**	-1.3	1.3

#### Gender x nation

The same test was conducted to examine an association between nation and gender. A significant result was found X^2^ (3) 1374.095 p < .001 and the association was very small [11] Cramer’s V = .01. Adjusted residuals can be viewed in Table 5.

**Table 5:** Chi^2^ test of independence adjusted residuals for nation with age groups and gender (analysed using dataset 1 –March 2017).

		England	Wales	Scotland	Northern Ireland	Unknown
**Gender**	**Male**	37.5	-5.8	-26.6	-21.5	-29.9
	**Female**	-37.5	5.8	26.6	21.5	29.9
**Registration Age Groups**	**0–4**	-54.4	-1.8	123.5	-92.6	4.7
	**5–9**	-10.7	1.7	45.2	-58.1	4.6
	**10–14**	-15.2	13.0	32.4	-44.7	20.3
	**15–19**	16.5	8.9	-21.8	5.6	-51.8
	**20–24**	-48.9	2.6	70.7	-22.9	20.0
	**25–29**	-33.6	-36.3	16.2	88.5	12.9
	**30–34**	-6.0	-36.9	13.4	31.4	16.0
	**35–39**	-33.5	-16.2	1.2	91.0	7.0
	**40–44**	-13.1	2.9	-2.2	29.8	-.4
	**45–49**	-1.7	13.0	-10.9	6.7	-.4
	**50–54**	13.6	7.7	-16.2	-10.7	-4.3
	**55–59**	21.4	8.6	-20.4	-23.5	.8
	**60–64**	28.8	8.0	-24.0	-33.0	.3
	**65–69**	96.1	14.3	-76.0	-95.5	-17.4
	**70–74**	46.0	30.6	-42.0	-68.8	2.1
	**75–79**	28.9	35.6	-42.6	-39.0	13.0
	**80–84**	8.0	29.7	-26.5	-18.1	29.4
	**85–89**	-2.6	20.7	-12.6	-6.8	28.0
	**90+**	25.1	-8.5	-27.4	.2	6.8
**Current Age Groups**	**0–4**	8.0	34.7	-7.0	-45.6	-12.0
	**5–9**	-46.1	-1.2	89.4	-50.5	-.9
	**10–14**	-47.7	-12.1	94.7	-44.6	5.1
	**15–19**	-40.4	5.4	24.8	50.1	-31.7
	**20–24**	-39.4	25.6	26.4	24.8	-48.9
	**25–29**	-26.8	-9.7	30.0	32.6	-41.7
	**30–34**	-29.2	-31.1	32.7	47.9	-1.6
	**35–39**	-2.6	-47.4	17.1	33.3	7.2
	**40–44**	8.2	-44.4	.5	32.4	13.2
	**45–49**	-.7	-18.3	9.6	4.3	11.6
	**50–54**	2.2	-7.2	.5	1.8	4.5
	**55–59**	-8.8	6.8	5.3	.6	3.9
	**60–64**	-11.8	22.3	2.8	-8.0	5.1
	**65–69**	16.3	31.7	-25.7	-34.2	9.1
	**70–74**	59.8	38.2	-67.9	-63.4	-.6
	**75–79**	53.5	32.3	-57.9	-60.3	2.5
	**80–84**	55.7	22.4	-58.2	-55.4	13.2
	**85–89**	49.8	18.7	-55.0	-44.9	18.8
	**90+**	9.4	58.6	-56.9	-26.9	103.6

#### Age x nation

Significant differences were also present for age groups and nation; registration age groups X^2^ (72) 93831.932 p < .001 and current age groups X^2^ (72) 97039.935 p < .001. The association was very small for both registration age [11] Cramer’s V = .03 and current age Cramer’s V = .03. Table 5 presents the adjusted residuals for these calculations.

#### Age x organ preference

A Chi^2^ test of independence was conducted for both registration age groups and current age groups with organ preference. Significant differences were found for all organs and registration age (Kidney X^2^(18)12262.802 p < .001; Pancreas X^2^(18) 9574.388 p < .001; Heart X^2^(8)76543.312 p < .001; Lungs X^2^(18) 12536.710 p < .001; Liver X^2^(18) 12259.513 p < .001; Cornea X^2^(18) 263085.991 p < .001). The associations were very weak for all organs except cornea ([11]; Cramer’s V kidney V = .02, Pancreas V = .02, Heart V = .06; Lungs V = .02, Liver V = .02). The association for cornea in contrast was of note, Cramer’s V = .11.

The tests were also all significant for all organs and current age (Kidney X^2^(18) 10815.178 p < .001; Pancreas X^2^(18) 8710.820 p < .001; Heart X^2^(18) 99566.938 p < .001; Lungs X^2^(18) 13518.615 p < .001; Liver X^2^(18) 11308.283 p < .001; Cornea X^2^(18) 366741.490 p < .001). As with registration age the associations were very weak for all organs except cornea ([11]; Cramer’s V Kidney V = .02, Pancreas V = .02, Heart V = .07; Lungs V = .03, Liver V = .02). The association for cornea in contrast was of note, Cramer’s V = .13. Table 6 shows where these significant differences lie.

**Table 6:** Chi^22^ test of independence adjusted residuals for saying yes to donating specific organs and age groups, gender and nation (analysed using dataset 1 –March 2017).

		KIDNEY	PANCREAS	HEART	LUNGS	LIVER	CORNEA
**Gender**	**Male**	-38	8.1	88.6	10.1	-25.7	433
	**Female**	38	-8.1	-88.6	-10.1	25.7	-433
**Current Age Groups**	**0–4**	19.0	30.4	28.5	32.2	26.2	17.4
	**5–9**	19.1	28.3	32.2	33.1	27.3	12.1
	**10–14**	14.9	17.4	28.5	27.4	22.6	4.4
	**15–19**	4.1	7.9	-54.4	-1.7	8.8	-94.7
	**20–24**	-9.4	-10.6	-154.9	-39.4	-10.4	-230.4
	**25–29**	-29.5	-22.4	-162.3	-55.5	-33.9	-248.3
	**30–34**	-32.5	-28.0	-129.5	-36.9	-41.6	-144.6
	**35–39**	-19.4	-18.8	-18.0	-15.8	-14.3	-44.5
	**40–44**	-4.3	-6.9	44.2	10.1	9.8	-29.6
	**45–49**	16.2	-4.0	68.0	16.9	28.8	-81.9
	**50–54**	27.7	-14.6	61.5	2.9	14.5	-37.2
	**55–59**	30.9	7.7	74.1	16.8	18.2	61.7
	**60–64**	35.0	38.0	87.1	40.3	31.5	153.8
	**65–69**	31.7	43.7	80.7	42.6	29.0	212.0
	**70–74**	10.0	35.1	61.5	28.9	11.5	243.4
	**75–79**	-17.0	.1	24.6	-1.7	-19.4	199.3
	**80–84**	-34.1	-13.4	8.1	-12.0	-33.3	170.5
	**85–89**	-35.7	-14.2	4.6	-10.9	-34.1	136.8
	**90+**	-31.4	-5.8	11.8	-1.8	-25.0	114.2
**Registration Age Groups**	**0–4**	31.0	34.6	51.8	49.7	44.2	15.3
	**5–9**	15.0	12.5	22.1	24.6	22.1	-2.2
	**10–14**	8.4	7.8	-6.3	14.5	15.6	-30.1
	**15–19**	-10.6	2.4	-221.3	-35.1	-23.2	-249.5
	**20–24**	-34.9	-41.0	-82.6	-46.5	-26.1	-161.8
	**25–29**	-13.3	-23.7	-13.6	-20.2	-5.4	-120.0
	**30–34**	5.3	-18.4	32.6	-4.7	4.6	-57.3
	**35–39**	17.6	-3.9	56.6	10.1	14.2	-14.4
	**40–44**	22.7	10.9	68.6	19.7	19.8	23.7
	**45–49**	27.2	20.4	75.1	26.7	21.0	73.9
	**50–54**	25.6	26.1	73.5	28.2	20.8	129.8
	**55–59**	19.3	32.0	66.9	30.9	18.0	171.7
	**60–64**	8.6	26.8	54.0	25.4	12.3	183.7
	**65–69**	-12.8	14.5	38.0	11.2	-10.7	228.3
	**70–74**	-43.1	-21.4	2.4	-20.5	-43.4	162.4
	**75–79**	-54.0	-37.4	-15.8	-35.3	-53.7	101.8
	**80–84**	-40.8	-27.5	-9.9	-24.9	-38.2	55.4
	**85–89**	-25.4	-14.9	-5.8	-13.1	-20.1	30.9
	**90+**	2.3	6.3	12.4	5.3	5.0	18.1
**Nation**	**England**	-28.3	-12.2	-42.9	-31.0	-30.0	62.9
	**Wales**	19.8	28.5	14.5	24.5	24.1	6.3
	**Scotland**	18.7	18.9	41.4	34.4	28.0	-46.8
	**Northern Ireland**	5.4	-40.3	5.3	-21.9	-12.4	-66.7

#### Nation x organ preference

Also found were significant results for nation and organ preference and the associations were all very weak. (Kidney X^2^(3)862.936 p < .001, Cramer’s V = 0.01; Pancreas X^2^(3) 2694.460 p < .001, Cramer’s V = 0.01; Heart X^2^(3) 2099.167 p < .001, Cramer’s V = 0.01; Lungs X^2^(3)2273.086 p < .001 Cramer’s V = 0.01; Liver X^2^(3) 1574.478 p < .001, Cramer’s V = 0.01; Cornea X^2^(3) 7038.209 p < .001, Cramer’s V 0.02). The adjusted residuals for these results can be viewed in Table 6.

#### Gender x organ preference

The final Chi^2^ tests of independence were conducted for organ preference with gender (adjusted residuals can be viewed in Table 6). Significant results were found for Kidney X^2^(1)1444.351 p < .001, Pancreas X^2^(1) 65.270 p < .001, Heart X^2^(1) 7858.002 p < .001, Lungs X^2^(1) 101.290 p < .001, Liver X^2^(1)658.992 p < .001 and Corneas X^2^(1) 187459.585 p < .001. The associations for all organs were very weak except Cornea ([11]; Kidney V = .01, Pancreas V = < .01, Heart V = .02, Lungs V = < .01), however the association strength for Cornea was close to the threshold of note V = .90 (Cohen, 1988).

#### Ethnicity x gender

As with the current dataset, Chi^2^ test of independence was conducted for ethnicity and gender X^2^(5) 2314.879 p < .001, the association was very weak [11] Cramer’s V = .02. Table 7 shows the adjusted residuals for this analysis.

**Table 7:** Chi^2^ test of independence adjusted residuals for ethnicity and gender (analysed using dataset 2 –April 2015).

		Gender	
		Male	Female
**Ethnicity**	**White**	-12.5	12.5
	**Asian**	41.9	-41.9
	**Black**	-5.2	5.2
	**Chinese**	-13.4	13.4
	**Mixed**	-19.5	19.5
	**Other**	1.4	-1.4

#### Ethnicity x age

Ethnicity and both current and registration age also produced significant results (current age: X^2^(90) 38825.694 p < .001, Cramer’s V 0.4; registration age: X^2^(90) 27063.798 p < .001, Cramer’s V 0.3) the adjusted residuals for these can be viewed in Table 8 and Table 9.

**Table 8:** Chi^2^ test of independence adjusted residuals for registration age group and ethnicity (analysed using dataset 2 –April 2015).

		Registration Age Groups																		
		0–4	5–9	10–14	15–19	20–24	25–29	30–34	35–39	40–44	45–49	50–54	55–59	60–64	65–69	70–74	75–79	80–84	85–89	90+
**Ethnicity**	**White**	-8.6	-4.6	-5.5	-36.2	-58.9	-42.4	-26.4	-7.2	13.9	32.7	42.3	44.9	44.4	42.6	21.3	12.2	7.8	4.5	-5.7
	**Asian**	-12.0	-11.6	-13.0	-11.9	25.9	33.6	23.7	10.4	-5.8	-16.0	-17.2	-14.5	-14.2	-14.2	-2.2	.5	.4	-.1	5.1
	**Black**	-5.9	-5.9	-5.4	-.4	22.3	14.5	19.7	14.9	10.3	-2.7	-14.5	-23.7	-25.5	-22.3	-12.9	-7.6	-5.6	-3.9	1.7
	**Chinese**	-2.6	-4.2	-3.2	13.2	29.0	12.7	4.3	-2.9	-7.5	-9.9	-10.6	-11.5	-11.6	-13.5	-8.8	-5.6	-3.2	-2.1	1.9
	**Mixed**	33.8	27.2	30.4	76.2	43.1	13.3	-1.5	-11.0	-21.9	-30.4	-36.4	-37.4	-36.3	-34.3	-21.6	-13.5	-8.9	-4.4	.8
	**Other**	-.6	-1.3	-2.9	-10.2	8.7	16.9	13.3	7.1	1.8	-5.0	-8.6	-9.7	-8.2	-8.4	-3.1	-2.8	-.6	.2	3.4

**Table 9:** Chi^2^ test of independence adjusted residuals for ethnicity and current age group (analysed using dataset 2 –April 2015).

		Current Age Groups																		
		0–4	5–9	10–14	15–19	20–24	25–29	30–34	35–39	40–44	45–49	50–54	55–59	60–64	65–69	70–74	75–79	80–84	85–89	90+
**Ethnicity**	**White**	-8.7	-9.5	-10.9	-43.7	-55.7	-64.7	-61.2	-52.7	-11.2	8.7	25.3	36.7	49.3	67.6	55.2	41.8	31.4	25.3	16.1
	**Asian**	-2.4	-6.9	-5.4	5.5	10.6	27.9	39.8	46.0	14.7	-9.3	-21.4	-16.6	-18.3	-32.9	-25.9	-17.9	-13.1	-13.3	-9.3
	**Black**	-1.2	-3.3	-2.9	10.1	16.2	17.1	18.7	18.0	8.3	16.3	12.3	-10.6	-26.5	-34.7	-27.7	-21.6	-14.8	-12.2	-6.8
	**Chinese**	-1.3	-2.3	-2.1	8.1	17.4	24.1	24.0	13.6	-.2	-8.5	-9.3	-10.0	-11.6	-15.2	-16.8	-11.8	-9.7	-7.2	-4.0
	**Mixed**	18.7	27.2	26.6	58.9	66.4	56.9	32.9	14.8	-6.2	-11.9	-21.6	-31.1	-38.7	-45.3	-37.1	-30.4	-23.1	-17.0	-10.6
	**Other**	.9	.3	2.6	1.8	2.0	6.4	14.0	15.2	7.5	.9	-3.9	-6.5	-9.7	-13.6	-10.3	-7.2	-7.0	-2.0	-1.2

## Discussion

The present study analysed the NHS Organ Donation Register (NHS ODR) to identify which demographic groups are over or underrepresented. The variables gender, age, ethnicity, nation and organ preference were analysed in two ways; using Chi^2^ Goodness of Fit and Chi^2^ Test of Independence. The former examined how the demographic patterns in the U.K. general population compare to the members of the NHS ODR and the latter compared variation between the demographic variables themselves.

All Chi^2^ Goodness of Fit analyses returned significant results, indicating the demographic patterns on the NHS ODR were all significantly different than those present in the U.K. population. However, Chi^2^ tests are sensitive to sample size and further tests are required to examine the strength of these results, namely using Cramer’s V. Only, ethnicity, registration age group and current age group had a Cramer’s V over .1 which indicates a small association strength according to Cohen [11].

Comparing ethnicity on the NHS ODR to the entire U.K., significantly more people identifying as White were registered (45,564) compared to Asian (-23,060), Black (-14,684), Chinese (-2,840), Mixed (-1571) or Other (-3410). The same pattern is present in England, for which a Cramer’s V of over .1 was found (White 42,145; Asian -21,486; Black -14,066; Chinese -2,178; Mixed -1,838; Other -2,577). However, for Wales, Scotland and Northern Ireland Cramer’s V did not reach the .1 threshold and therefore the significant result was not of note.

For registration age, the biggest difference between age groups on the NHS ODR compared to the U.K. population was found for 15–19 year olds with 2,954,396 more people registering than expected for this age group. There were also more 20–24 (882,081), 25–29 (1,277,626), 30–34 (865,403), 35–39 (475,534) and 40–44 year olds (61,114). Also of note is the lack of under 15’s registering compared to the general population, 1,253,119 less 0–4 year olds, 1,135,776 less 5–9 year olds and 1,101,976 less 10–14 year olds than expected. With regard to the older age groups at registration all groups above the age of 45 had less than expected registrations (45–49; -109,581; 50–54–166,336; 55–59–249,111; 60–64–559,520; 65–69–135,826; 70–74–373,964; 75–79–504,701; 80–84–478,292; 85–89–323,876; 90+ -124,072).

For current age, less 0–14 year olds are on the ODR than expected (0–4–1,373,651; 5–9–1,198,325; 10–14–1,246,285) however there are also less 15–24 year olds than expected currently on the register (15–19–878,777; 20–24–117,908). There are also less older people registered on the NHS ODR than expected in the age groups 60–64 (-8,641), 80–84 (-36,870) and 85–89 (-39,067). The age groups where there are more people than expected on the NHS ODR are 25–29 (442,559), 30–34 (738,117), 35–39 (930,580), 40–44 (611,656), 45–49 (714,323), 50–54 (638,926), 55–59 (400,841), 65–69 (134,439), 70–74 (253,553) and 75–79 (11,059)

Gender and Nation both had Cramer’s V under .1, therefore although Chi^2^ was significant the result did not reach the adequate strength of association. However, 658,722 more women than men were present on the register and 583,105 less people than expected were on the register from England compared with 61,064 more in Wales, 451,947 more in Scotland and 70,093 more in Northern Ireland.

These results help examine which groups are over and under represented on the ODR. By examining the strength of the Chi^2^ test, the importance of these patterns can be used to target future campaigns. Compared to the general U.K. population, age group and ethnicity should both be a focus for campaigns due to their Cramer’s V value above .1 as a priority over gender and nation.

The issue of a lack of representation of Black, Asian and Minority Ethnic groups on the NHS ODR has been discussed at length [10,14]. Reasons for the lack of sign up in these groups include; lower donation knowledge, less likely to discuss donation and their wishes with family members, unacceptable due to religious beliefs and a lack of trust in medical professionals [15]. However, no study has yet examined statistically where this underrepresentation appears in comparison to the U.K. population. Previous studies have examined interventions specifically targeting ethnic minorities [16], and suggested that community level interventions specifically targeting those who are underrepresented are more successful than mass media campaigns. This was suggested to be due to the tailoring to the specific concerns of the ethnic group, rather than generalized concerns present in the general population [16]. The current study suggests that people of Asian ethnicity should be specifically targeted first, followed by those of Black ethnicity. The results of the review by Deedat et al [16] suggest tailoring to these groups using community interventions could improve registration rates. It is interesting that these results are only of notable strength for England, whereas the Cramer’s V for Wales, Scotland and Northern Ireland are less than .1. This could be due to the lower proportion of BAME ethnicity in these countries than in England [17]. However, it would be interesting to examine if nation specific BAME campaigns could be responsible for this difference between nations.

Age representation on donor registries has received less attention in the literature, however NHSBT do highlight a need to increase registrations made by those over 50 years of age [1]. Additionally the higher level of those aged 15–19 at registration, could be due to access to NHS ODR sign-up at through the Driver and Vehicle Licensing Agency [1]. At age 15 years and 9 months in the U.K. adolescents are able to apply for their provisional driving licenses and will be given the option to register on the NHS ODR through this process [18]. In 2017–2018 50% of registrations were made by the DVLA, the most common method of sign-up in the U.K. suggesting that access to NHS ODR sign-up through the DVLA could be responsible for the peak in this age group [1]. A recommendation for future exploration would be to examine how demographic variables interact with source of registration.

When interpreting results concerning age, it is important to examine the limits of age on donation. The NHSBT ODR website states that children can join the ODR however parental consent must be given after they die for their organs to be donated. A lack of knowledge of this could be responsible for the less than expected representation of those under 15 on the ODR. Additionally, the only age restrictions on actual donation are no heart valve or tendon donations are permitted after the age of 60 and no cornea donations after the age of 80 years old [19].In contrast to these actual restrictions, a study in England examined beliefs toward organ donation and found that a belief participants were “too old” acted as a barrier towards registering on the ODR [20]. Examining the results of the present study however, there are less people than expected on the ODR currently aged 15–24 and more than expected present from age 25–79 (except age group 60–64) which is in contrast to the NHSBT specification to target those over 50 and the results of Webb et al’s study (2015). These results are interesting when examined with the age at which people register on the NHS ODR, with less people registering from the ages of 45–90+. An explanation for this could be a higher number of people registered when they were in the younger age groups, who are now older. The less than expected presence on the ODR of those aged 15–24 indicates that efforts to recruit younger people have been less successful in recent years and emphasis should be placed on recruiting people currently aged 15–24.

These findings are of particular importance when comparing them to the demographic characteristics of actual organ donors. In 2016–2017, 57% of actual donors were male, compared with making up 46.5% of the NHS ODR. 83.3% of actual donors were from England, 4.3% from Wales, 9.4% from Scotland, 3% from Northern Ireland; compared with 81.8%, 5.0%, 10.1% and 3.1% on the ODR respectively. Although the strength of the association was weak in comparison to the population, it is useful to consider that if there were more English and male actual donors in 2016–2017, it may indicate a need to increase ODR membership of those demographics to be closer to the prevalence of actual donors. When comparing age groups of actual donors to those on the ODR, more actual donors were aged 70+ (14%), 60–69 (22%), 50–59 (24%) than on the ODR (70+ = 4%; 60–69 = 7.8%; 50–59 = 10.4%). This indicates that the age profile of the NHS ODR favoring these older groups could be influential in these results. The percentage of actual donors over 60 years of age has increased from 21% to 36% since 2007–2009 and this could reflect the ageing nature of the NHS ODR. It is important however to note this is merely speculation, further research is required to investigate the cause and effect of this shift to older donors and the influence historic donation campaigns has had on ODR membership.

To help explore these findings, the second set of analyses were conducted to investigate intra-demographic patterns. Chi^2^ test of independence was used to examine associations between variables. As with the above Chi^2^ goodness of fit analyses all were significant, however not all analyses reached the Cramer’s V threshold of .1 indicating association strength.

For both registration and current age groups, only the analyses examining age and Cornea donation preference had a Cramer’s V over .1. Adjusted residuals were examined with those higher than 2.0 or -2.0 considered significant. At registration age, the older age groups (except Under11, AR 10.7), 41–50 (AR 85.5), 51–60 (AR 229.5), 61–70 (AR 304.7), 70+ (AR 172.3) were significantly more likely to donate their corneas than the younger age groups 11–15 (AR-15.8), 16–20 (AR-261.2), 21–30 (AR-195.6), 31–40 (AR -41.4). When examining this result for current age a similar pattern is present with older groups (except under 11 AR 20.8) significantly more likely to donate their corneas than younger groups (16–20 AR -129.3; 21–30 AR -354.8; 31–40 AR -111.9, 41–50 AR -92.2, 51–60 AR 42.6, 61–70 AR 289.1, 70+ AR398.2). Examining the results for the variables nation and gender, no result reached the Cramer’s V value of .1 however the analyses examining gender with cornea preference was near this threshold at .9. Significantly more men than women would elect to donate their cornea (AR 433).

In September 2017 NHSBT specified that eye banks responsible for the storage of donated corneas were running at 21% below the level required to meet demand [21]. In the 2016–2017 activity report of those who chose to select which organs they donated, 90% were not willing to donate their corneas. Of all registrants this makes up a 10.7% refusal rate [1]. Studies have examined the predictors of cornea donation and found several factors which could provide a barrier to corneal donation. A lack of knowledge [22], concerns about disfigurement [22–25], that eyes are associated with the soul [26,27] and that sight is required in the afterlife [28]. The patterns found in the present study, that more male and older people are selecting to donate their corneas can be used to target younger female groups and addressing the barriers found in previous research. However, the evidence on these barriers is limited to studies conducted over 10 years ago or using small samples.

As previously stated, the topic of ethnicity and ODR membership has received a lot of attention. There is a larger discrepancy between the number of BAME people waiting for a transplant and those donating their organs [10]. For all Chi^2^ tests of association with ethnicity and the other variables in the dataset, none of these reached the .1 threshold for Cramer’s V. This shows that the underrepresentation of BAME groups on the ODR is not associated with gender or age. Unfortunately, due to the removal of ethnicity from the main ODR dataset as described previously, an examination between ethnicity and organ preference could not be conducted.

When interpreting these results, it is important to be mindful of a few limitations. Although the missing data made up less than 1%, this equated to 7334 missing current ages, 206068 registration ages, 67308 missing gender and 64381 missing nation. Although small in proportion to the total N for the dataset, some residuals were smaller than the number of missing values. Therefore, conclusions made should be treated with caution as the true values of each demographic category could be significantly different from that recorded. An additional limitation comes from the descriptive nature of this study. Although important to further our understanding the demographic profile of the NHS ODR, the results are descriptive and inferences of causality cannot be made. Only inferences of why certain groups may be over or underrepresented are made. It is also important to specify that the variables analysed are only those provided by NHSBT for analysis. There could be other potential influential variables which could explain ODR membership.

In spite of limitations, these results provide practical implications for future organ donation campaigns which target NHS ODR membership. Previous NHS BT activity reports have highlighted the shortage of older people registering on the NHS ODR [1]. The strength of association found in the present study contributes to this highlighting age discrepancies are an area that receive attention, that older people are less likely to register. A pervasive myth concerning organ donation, that creates a barrier to registration sign-up is that a person is “too old” or that their organs will not be usable due to their age or health reasons [29–31]. Downing & Jones [30] designed and implemented an intervention formed of an educational brochure specifically targeting older adults aged 50+. This was based on a survey and focus group with older adults in Ohio. They found 48% of older adults who would not donate their organs stated this was due to age or health related reasons [30]. In contrast to these beliefs however, was the higher levels of positive attitude towards donation itself in the older age groups compared with younger age groups. Although a study based in the U.S.A, these results help provide guidance on how exactly older age groups could be targeted in the U.K.; through combatting incorrect beliefs concerning eligibility to donate.

A challenging finding from this study is the underrepresentation of 0–14 year olds at registration and also currently on the NHS ODR. Children of any age can join the ODR, however consent to donate their organs ultimately lies with their parents until the age of 16 or 18 depending on where they are located [19]. Intervention specifically targeting children is less prevalent than those targeting adults [32]. A series of studies in the Netherlands examined the targeting of primary school aged children to encourage organ donation discussion [33–36]. These studies showed 99% of children aged 12–15 were aware of organ donation [34]; 46.5% of parents had discussed organ donation with their children (N1146; [35]); 70% of teachers were in favour of having lessons on organ donation, with the best age suggested to be age 10–11 [36]; and 20% of children who took part in an educational lesson reported having further discussions with their parents on the subject [35]. These studies show promise for intervention to encourage family discussion of organ donation with children over the age of 10. An additional component in these educational interventions could be encouraging registration on the NHS ODR, alongside the encouragement of discussion. However, the question still arises, is ODR membership a necessary addition if parents make the final decision on donating their child’s organs? Or is simply encouraging discussion within family units enough? These are questions which require further consideration by U.K. intervention developers, ethicists and NHS Blood and Transplant.

Finally, it is important to discuss that whilst undertaking this research, the U.K. government opened a consultation on moving to an opt-out system in England from an opt-in system [37], the findings of which subsequently confirmed that England will move to opt-out as of April 2020 [38]. The system will mirror the one employed in Wales, whereby family will still be consulted regarding donation wishes [39]. Previous transitions to an opt-out system e.g. in Spain have found that opt-out on its own did little to increase donation rate, primarily due to family consent still being prevalent in the process [40]. Importantly, in Spain no opt-out register is present and public knowledge of the deemed consent law is minimal [40]. Further, a recent study found that people were more likely to refuse to donate their loved ones organs in an opt-out system than in an opt-in [39]. Examining the impact of opt-out versus opt-in systems internationally, Shepherd, O’Carroll and Ferguson (2014) [41] found that opt-out systems have higher rates of deceased organ donation. However, they also state that assuming opt-out is responsible for this increase is too simplistic, particularly as opt-out decreased donation rates in some countries (France and Brazil). Although some increase in actual donation rate is expected, it is still important that opt-out is not treated as a complete fix for increasing rates of organ donation in the U.K. In depth analysis on underrepresented groups and targeted campaigning can still go ahead during the transition to the new system and following its introduction. It will still remain a vital part of increasing rates of organ donation through active registration on the NHS ODR.

In summary, our study shows the demographic patterns of age group, nation and gender present in the NHS ODR are significantly different from the demographic patterns of the U.K. Age group at registration, current age group and ethnicity however are the only variables with a strength of association worth examining. These variables on the ODR are significantly different from the age groups and ethnicity in the U.K. population. Registration age groups of 0–14 and 45+ and current age groups of 0–19 and 80+ are underrepresented on the NHS ODR as are those of BAME ethnicity. These findings suggest targeting campaigns to both the older and younger age groups in the U.K. population to increase their membership of the NHS ODR.
