# Correction: Who's opting-in? A demographic analysis of the U.K. NHS Organ Donor Register

**DOI:** 10.1371/journal.pone.0213628

**Published:** 2019-03-06

**Authors:** Catrin Pedder Jones, Chris Papadopoulos, Gurch Randhawa

An incorrect Cramer’s V value appears twice in the article. The value appears as .90; the correct value is .09.

As a result, in the Tests of association subsection of the Results, the last sentence of the “Gender x organ preference” paragraph is incorrect. The correct sentence is: The associations for all organs were very weak except Cornea ([11]; Kidney V = .01, Pancreas V = < .01, Heart V = .02, Lungs V = < .01), however the association strength for Cornea was close to the threshold of note V = .09 (Cohen, 1988).

In addition, in the Discussion section, the penultimate sentence of the 13th paragraph is incorrect. The correct sentence is: Examining the results for the variables nation and gender, no result reached the Cramer’s V value of .1 however the analyses examining gender with cornea preference was near this threshold at .09.
